# Degradation of malachite green by UV/H_2_O_2_ and UV/H_2_O_2_/Fe^2+^ processes: kinetics and mechanism

**DOI:** 10.3389/fchem.2024.1467438

**Published:** 2024-10-24

**Authors:** Sumaira Wilayat, Perveen Fazil, Javed Ali Khan, Amir Zada, Muhammad Ishaq Ali Shah, Abdulaziz Al-Anazi, Noor S. Shah, Changseok Han, Muhammad Ateeq

**Affiliations:** ^1^ Department of Chemistry, Abdul Wali Khan University Mardan, Mardan, Khyber Pakhtunkhwa, Pakistan; ^2^ Department of Chemistry, University of Karachi, Karachi, Pakistan; ^3^ UNESCO-UNISA Africa Chair in Nanosciences and Nanotechnology, College of Graduate Studies, University of South Africa, Muckleneuk Ridge, Pretoria, South Africa; ^4^ Department of Chemical Engineering, College of Engineering, King Saud University, Riyadh, Saudi Arabia; ^5^ Department of Chemistry, COMSATS University Islamabad, Abbottabad, Pakistan; ^6^ Program in Environmental and Polymer Engineering, Graduate School of INHA University, Incheon, Republic of Korea; ^7^ Department of Environmental Engineering, INHA University, Incheon, Republic of Korea

**Keywords:** malachite green, UV light, hydrogen peroxide, photo-Fenton, degradation mechanism, wastewater treatment

## Abstract

This work investigated the photochemical degradation of malachite green (MG), a cationic triphenylmethane dye used as a coloring agent, fungicide, and antiseptic. UV photolysis was ineffective in the removal of MG as only 12.35% degradation of MG (10 mg/L) was achieved after 60 min of irradiation. In contrast, 100.00% degradation of MG (10 mg/L) was observed after 60 min of irradiation in the presence of 10 mM H_2_O_2_ by UV/H_2_O_2_ at pH 6.0. Similarly, complete removal (100.00%) of MG was observed at 30 min of the reaction time by UV/H_2_O_2_/Fe^2+^ employing [MG]_0_ = 10 mg/L, [H_2_O_2_]_0_ = 10 mM, [Fe^2+^]_0_ = 2.5 mg/L, and [pH]_0_ = 3.0. For the UV/H_2_O_2_ process, the degradation efficiency was higher at pH 6.0 than at pH 3.0 as the *k*_obs_ values were 0.0873 and 0.0690 min^−1^, respectively. However, UV/H_2_O_2_/Fe^2+^ showed higher reactivity at pH 3.0 than at pH 6.0. Chloride and nitrate ions slightly inhibited the removal efficiency of MG by both UV/H_2_O_2_ and UV/H_2_O_2_/Fe^2+^ processes. Moreover, three degradation products (DPs) of MG, (i) 4-dimethylamino-benzophenone (DABP), (ii) 4-amino-benzophenone (ABP), and (iii) 4-dimethylamino-phenol (DAP), were identified by GC-MS during the UV/H_2_O_2_ treatment. These DPs were found to demonstrate higher aquatic toxicity than the parent MG, suggesting that researchers should focus on the removal of target pollutants as well as their DPs. Nevertheless, the results of this study indicate that both UV/H_2_O_2_ and UV/H_2_O_2_/Fe^2+^ processes could be implemented to alleviate the harmful environmental impacts of dye and textile industries.

## 1 Introduction

Synthetic dyes are primarily used in the textile, food, and cosmetics industries. These industries frequently discharge a large amount of untreated and partially treated dye effluents, leading to substantial environmental pollution (Slama et al., 2021). These dyes pose significant harm to human beings and other ecosystems. According to available literature, certain synthetic dyes exhibit toxic properties such as dermatologic effects, allergenic, and carcinogenic (Arora, 2014; Tkaczyk et al., 2020). These dyes constitute the largest category of all colorants, with more than 100,000 varieties available commercially worldwide. The global production of synthetic dyes exceeds 1 million tons annually (Tkaczyk et al., 2020). The extensive use of dyes across various fields greatly impacts water sources (Slama et al., 2021; Tkaczyk et al., 2020; Lal et al., 2024). Synthetic dyes are extensively used in the textile, leather, printing, and paper industries, with certain varieties also being applied in the pharmaceutical and cosmetics industries and in food production (Tkaczyk et al., 2020). The large-scale production of dyes and their broad range of applications lead to the generation of large volumes of colored wastewater and various types of post-production wastes. The textile industry accounts for a significant amount of dyes in aquatic environments, with dye losses during dyeing processes ranging from a minimum of 5% to as much as 50%, depending on the type of fabric and dye. As a result, approximately 200 billion liters of colored effluents are produced annually (Kant, 2012). Additionally, the discharge of textile chemicals raises concerns and presents scientific challenges (Kishor et al., 2021). Given their commercial value, the impacts and risks associated with these chemicals have been studied intensively (Katheresan et al., 2018).

Malachite green (MG), a synthetic dye, is commonly used in aquaculture, textile, and food product industries (Sharma et al., 2023). MG poses potential risks to aquatic life, human health, and the environment (Sharma et al., 2023; Gharavi-Nakhjavani et al., 2023). As a result, regulatory bodies worldwide have taken measures to restrict or ban its use (Gopinathan et al., 2015). Therefore, it is essential to efficiently eliminate synthetic organic dyes from (waste) water (Singh and Arora, 2011). In this regard, different methods have been explored. Several physical, biological, and chemical methods have been used for this purpose, including adhesion to substances, chemical precipitation, photochemical and/or chemical degradation (Khan I. et al., 2020), adsorption (Lanjwani et al., 2024), coagulation (Khan I. et al., 2020; Lanjwani et al., 2024), membrane processes (Oladoye et al., 2023), as well as microbial decolorization (Alsukaibi, 2022) or biological degradation (Thao et al., 2023). Traditional methods were insufficient to treat wastewater containing these stable toxins. However, some methods are very effective in the removal/degradation of toxic environmental pollutants (Sivaraman et al., 2022; Mishra et al., 2019), among which advanced oxidation processes (AOPs) are predominant (Garrido-Cardenas et al., 2020; Fast et al., 2017).

AOPs use highly reactive oxidizing species such as ^•^OH and SO_4_^•‒^ to remove organic pollutants from water bodies (Khan et al., 2013; Islam et al., 2023; Hu et al., 2022; Sepúlveda et al., 2024). AOPs have the advantages of being environmentally friendly, achieving complete degradation of pollutants (Fast et al., 2017), and being relatively low cost compared to other methods. Additionally, AOPs can be applied on-site, minimizing the need to transport and treat effluents. Different oxidants such as hydrogen peroxide (H_2_O_2_), peroxymonosulfate (HSO_5_^−^), and persulfate (S_2_O_8_^2‒^) are generally used in AOPs as sources of reactive radicals. Among different AOPs, UV/H_2_O_2_ and UV/H_2_O_2_/Fe^2+^ processes are commonly used as (waste)water treatment processes (Khan et al., 2014). Both UV/H_2_O_2_ and UV/H_2_O_2_/Fe^2+^ processes are based on the formation of highly reactive and non-selective hydroxyl radicals (^•^OH). The hydroxyl radical (^•^OH) possesses a standard redox potential (E^o^) of +2.80 V versus the normal hydrogen electrode (NHE). It exhibits a short lifetime (*t*_1/2_ < 1 μs) and high reactivity, making it a powerful oxidant. Moreover, the reactivity of hydroxyl radical is pH-dependent and has a typical reaction rate of 10^6^ to 10^11^ M^−1^ s^−1^ with organic compounds. Notably, ^•^OH reacts in a non-selective manner, engaging in various reactions including electron transfer, addition, and abstraction of hydrogen, impacting their reactivity and potential applications in pollutant removal and environmental remediation (Oh et al., 2016).

This study explores the efficacy of MG degradation by UV, UV/H_2_O_2_, and UV/H_2_O_2_/Fe^2+^ processes. The effect of different H_2_O_2_, MG, and Fe^2+^ concentrations and pH conditions on the removal efficiency of MG was evaluated. Moreover, the impact of nitrate and chloride ions was also investigated. In addition, the degradation products (DPs) of MG were identified, and potential degradation pathways were proposed. Finally, the toxicity of the identified DPs and MG was determined using the Ecological Structure Activity Relationship (ECOSAR) Program.

## 2 Experimental

### 2.1 Chemicals and reagents

Malachite green hydrochloride (C_23_H_25_N_2_Cl, molar mass = 364.911 g/mol), hydrochloric acid (HCl, 37.0%), sulfuric acid (H_2_SO_4_, 95.0%–98.0%), sodium hydroxide (NaOH, ≥97.0%), potassium chloride (KOH, ≥85.0%), and sodium nitrate (NaNO_3_, ≥99.0%) were purchased from Sigma-Aldrich. Hydrogen peroxide (H_2_O_2_, 30% v/v) and ferrous sulfate heptahydrate (FeSO_4_.7H_2_O) were supplied by Merck.

### 2.2 Experimental procedures

Experiments were conducted in a photo-reactor consisting of a 100-mL beaker, kept on a magnetic stirrer to continuously mix the reaction solution. The radiation source was a 35-Watt UV lamp (UV-C, wavelength 254 nm, manufactured by Philips, Holland). The concentration of MG in the irradiated solutions was 10 mg/L, if not stated otherwise. Samples of 5 mL from the irradiated solutions were collected at specific time intervals for analysis. Whenever needed, the pH was adjusted with HCl and NaOH.

### 2.3 Analytical methods

A UV–Vis spectrophotometer (PerkinElmer) was used to measure the MG concentration in the treated solutions. The absorbance was recorded at 617 nm. Gas chromatography–mass spectrometry (GC-MS, Agilent Technologies, 6890 Series) was used to identify the DPs of MG. In GC-MS, the separation was carried out with an HP-5MS capillary column (30 m, 0.25 mm I.D., 0.25 μm). The oven temperature was programmed as follows: 100°C (1 min) to 180°C at a rate of 20°C min^‒1^ (3 min) and finally set to 250°C at a rate of 10°C min^‒1^ (2 min). The temperatures of the injector and MS detector were set at 250°C and 280°C, respectively. The carrier gas was helium (flow rate = 1.0 mL min^−1^). The MS was applied in the EI mode at 70 eV. The DPs were determined at full scan mode ranging from 50 to 550 amu.

Equation 1 was used to determine the degrading efficiency of MG.

$$\text{Degradation efficiency }\left(\%\right)=\left[\left(C_{0}\;–\;C_{t}\right)\;/\;C_{0}\right]\times 100,$$ (1)

where C_0_ represents the starting concentration of MG and C_t_ represents the MG concentration at time t.

### 2.4 Frontier electron densities and point charge calculations

To find the reactive sites/centers in the MG molecule where ^•^OH can preferentially attack, the frontier electron densities (FEDs) of the molecular orbitals of MG and point charges of the C and N atoms were calculated using the Gaussian 09 program (An et al., 2015; Rehman et al., 2018). FED calculations were performed for both the highest occupied molecular orbitals (HOMOs) and the lowest unoccupied molecular orbitals (LUMOs). Initially, the geometry of MG was optimized using the HF/3–21 g basis set. Thereafter, the energy calculations for FEDs and point charge determination were performed using the hybrid density functional B3LYP method of the density functional theory (DFT) with the 6–311 g basis set (B3LYP/6–311 g).

### 2.5 Determination of aquatic toxicity

The Ecological Structure Activity Relationship (ECOSAR) program (ECOSAR, 2014) was used to evaluate the acute and chronic toxicities of MG and its DPs. The ECOSAR is an effective tool for predicting the aquatic toxicity of toxic pollutants (Ali et al., 2018). This program evaluates the acute and chronic toxicities of toxic compounds toward fish, daphnia, and green algae. Acute toxicity is evaluated in terms of the LC_50_ and EC_50_. LC_50_ is the toxicant concentration which could cause the death of 50% fish and 50% daphnia after 96 and 48 h of exposure, respectively. EC_50_ is the concentration of the toxicant responsible for 50% growth inhibition of green algae after 96 h of exposure.

## 3 Result and discussion

### 3.1 Direct photolysis of MG

Before investigating the efficiencies of UV/H_2_O_2_ and UV/H_2_O_2_/Fe^2+^ processes for the degradation of MG, it was studied by direct photolysis (UV only) employing UV-C (254 nm) light. The 254-nm UV-C light photons do not have sufficient amount of energy to directly split water molecules into reactive species, i.e., hydroxyl radicals (^•^OH) and hydrated electrons (e^−^_aq_), because only radiation with λ < 190 nm can split water to produce ^•^OH and e^−^_aq_ (Gonzalez et al., 2004; Imoberdorf and Mohseni, 2011). Therefore, any degradation of MG at 254 nm radiation can only be attributed to the formation of its excited-state species (MG*) upon absorption of 254 nm photons (reaction (Equation 2)) (Khan et al., 2014). The excited-state molecules can either undergo direct degradation (reaction (Equation 3)) or may lead to the formation of reactive oxygen species (singlet oxygen, ^1^O_2_) (reaction (Equation 4)) which then attack the MG molecules and cause their degradation (reaction (Equation 5)) (Chen et al., 2008).

MG+hv → MG*, (2)

$${\text{MG}}^{*}\to \text{Degradation products},$$ (3)

MG*+O32 → MG+O12, (4)

MG+O12→Degradation products. (5)

**FIGURE 1:**
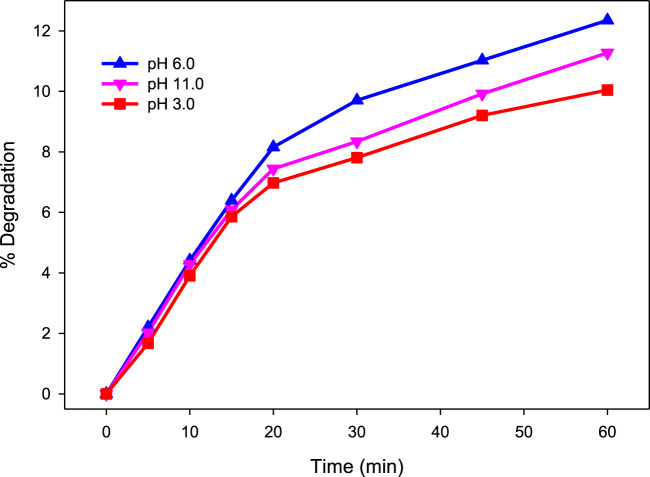
Effect of pH on % degradation of MG by direct photolysis. [MG]_0_ = 10 mg/L.

In this study, only 12.35% degradation of MG (10 mg/L) was achieved by direct photolysis at pH 6.0 after 60 min of irradiation. At pH 3.0 and 11.0, a slight decrease in degradation from 12.35% to 10.04% and 11.27%, respectively, was observed. The observed *pseudo-first-order* rate constant (*k*_obs_) was 0.0027, 0.0024, and 0.0022 min^−1^ at pH 6.0, 3.0, and 11.0, respectively. These results suggest that direct photolysis is not an effective method for the removal of MG from water (Figure 1). These results are in agreement with findings of Modirshahla and Behnajady (2006). Therefore, UV-C was accompanied by H_2_O_2_ and H_2_O_2_/Fe^2+^ in the following experiments for efficient degradation of MG.

### 3.2 Degradation of MG by UV/H_2_O_2_ and UV/H_2_O_2_/Fe^2+^ processes

#### 3.2.1 Effect of the initial H_2_O_2_ concentration

Both UV/H_2_O_2_ and UV/H_2_O_2_/Fe^2+^ processes are called hydroxyl radical-based AOPs since they result in the formation of ^•^OH, as shown in (Equations 6–9) (Khan et al., 2013; Khan et al., 2017; Shah et al., 2015; Jiad and Abbar, 2023).

H2O2+hv→2O•H ϕ=1.0, (6)

H2O2+Fe2+→O•H+OH‒+Fe3+, (7)

H2O2+Fe2+→O•H+FeIIIOH2+, (8)

FeIIIOH2++hv→O•H+Fe2+. (9)

The reaction (Equation 9) regenerates ferrous ions (catalyst) during the treatment process and thus helps in continuous production of ^•^OH in the reaction mixture, thereby degrading the target pollutants until their complete mineralization, as long as the source of ^•^OH (i.e., H_2_O_2_) is available in the solution.

**FIGURE 2:**
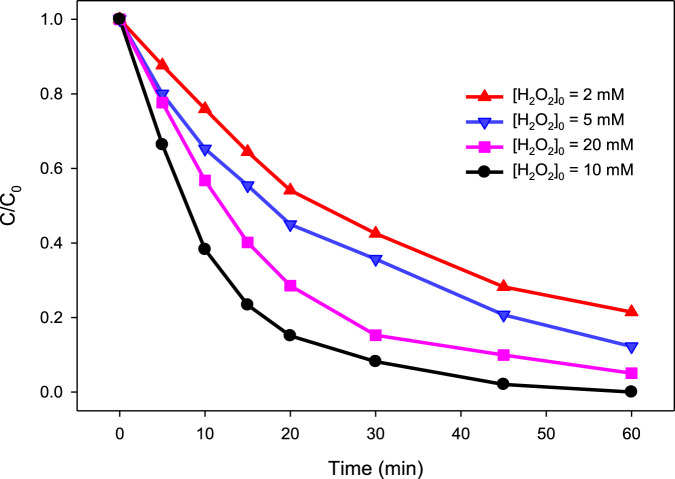
Effect of H_2_O_2_ concentration on % degradation of MG in the UV/H_2_O_2_ process. [H_2_O_2_]_0_ = 2 mM, 5 mM, 10 mM, and 20 mM, [MG]_0_ = 10 mg/L.

In the present study, four different concentrations of H_2_O_2_, i.e., 2, 5, 10, and 20 mM, were tested for their ability to degrade MG by the UV/H_2_O_2_ process at pH 6.0 using 10 mg/L MG. The results are depicted in Figure 2. It was observed that a combination of H_2_O_2_ with UV has significantly improved the degradation of MG compared to direct photolysis. The degradation of MG was found to be 78.5, 87.8 and 100% at 60 min in presence of 2, 5 and 10 mM H_2_O_2_, respectively, compared to 12.35% in direct photolysis. This increase in % degradation was due to the generation of ^•^OH in the UV/H_2_O_2_ process (reaction (Equation 6)). Since ^•^OH radicals are highly reactive and non-selective species, they reacted fast with MG, resulting in its degradation. As the concentration of H_2_O_2_ increases in the reaction solution, a larger pool of ^•^OH is expected to be produced, thereby enhancing the degradation process. However, a further increase in H_2_O_2_ concentration to 20 mM was found to have a detrimental effect on the degradation efficiency as only 94.9% degradation of MG was observed at the H_2_O_2_ concentration of 20 mM. The negative impact of higher H_2_O_2_ concentration on the degradation efficiency of MG was due to the scavenging effect of H_2_O_2_ for ^•^OH (Equation 10) (Khan et al., 2013; Modirshahla and Behnajady, 2006; Khan J. A. et al., 2020; Wei et al., 2020; Rauf et al., 2016). Since this scavenging effect is predominant at higher H_2_O_2_ concentration, a relatively lesser degradation of MG was observed at higher H_2_O_2_ concentration (20 mM) than the optimum value (10 mM). The HO_2_^•^ has been reported to be less reactive as compared to ^•^OH and hence do not contribute much toward MG degradation (Modirshahla and Behnajady, 2006; Hameed and Lee, 2009). The *k*_obs_ was calculated to be 0.0271, 0.0355, 0.0873, and 0.0529 min^−1^ at 2.0, 5.0, 10.0, and 20.0 mM H_2_O_2_, respectively. Therefore, it is crucial to optimize the H_2_O_2_ concentration to achieve the best result in pollutant removal in H_2_O_2_-assisted AOPs.

H2O2+O•H→H2O+HO2•. (10)

**FIGURE 3:**
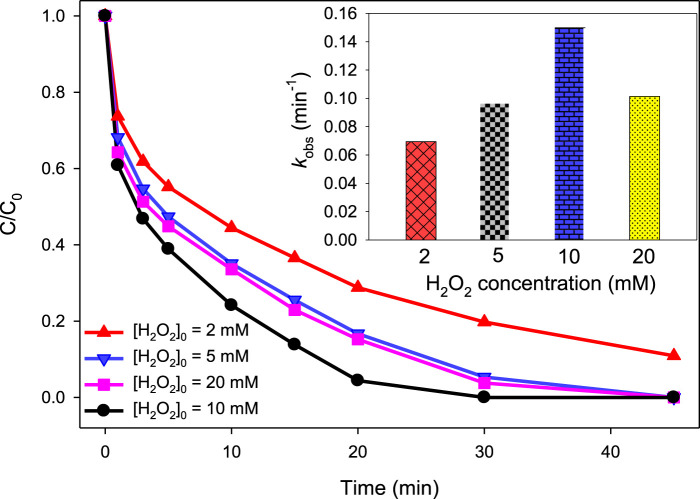
Effect of H_2_O_2_ concentration on the UV/H_2_O_2_/Fe^2+^ process. [Fe^2+^]_0_ = 2.5 mg/L, [MG]_0_ = 10 mg/L, [H_2_O_2_]_0_ = 2, 5, 10, and 20 mM, pH 3.0. The inset indicates the *k*_obs_ values at studied H_2_O_2_ concentrations.

In case of UV/H_2_O_2_/Fe^2+^, the same initial concentrations of H_2_O_2_ were studied as in the case of the UV/H_2_O_2_ process, i.e., 2, 5, 10, and 20 mM. The initial concentration of MG was 10 mg/L, that of Fe^2+^ was 2.5 mg/L, and the pH was 3.0. The purpose of acidic pH, i.e., pH 3, was to keep the photo-Fenton system highly efficient as the catalytic activity of Fe^2+^ for H_2_O_2_ activation has been found to be the highest at acidic pH values (Khan et al., 2013; Hameed and Lee, 2009; Nasuha et al., 2021). At an initial H_2_O_2_ concentration of 2, 5, 10, and 20 mM, the degradation efficiency was found to be 71.21, 83.31, 95.61%, and 84.77%, respectively, at 20 min (Figure 3). Similarly, the *k*_obs_ values at 2, 5, 10, and 20 mM H_2_O_2_ were found to be 0.0695, 0.0957, 0.1499, and 0.1014 min^‒1^, respectively. This trend is similar to that found in the UV/H_2_O_2_ process. Thus, it can be suggested that the role of H_2_O_2_ is similar in both UV/H_2_O_2_ and UV/H_2_O_2_/Fe^2+^ processes, i.e., initially increasing the level of ^•^OH up to a certain optimum concentration of H_2_O_2_ (10 mM in this case) through reactions Equations 6–9 and then decreasing the level of ^•^OH *via* the scavenging process (reaction (Equation 10)).

#### 3.2.2 Effect of initial MG concentration

**FIGURE 4:**
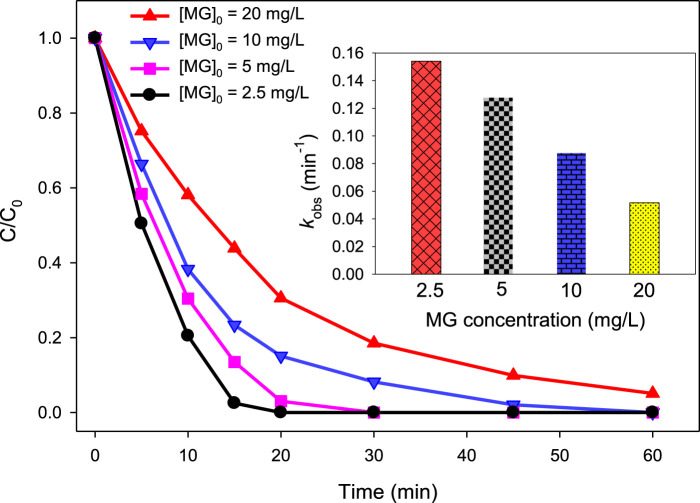
Effect of MG concentration on % degradation of malachite green in the UV/H_2_O_2_ process. [H_2_O_2_]_0_ = 10 mM, [MG]_0_ = 2.5 mg/L, 5 mg/L, 10 mg/L, and 20 mg/L. The inset indicates the *k*_obs_ values at different MG concentrations.

To analyze how varying concentrations of MG influence its degradation efficiency by UV/H_2_O_2_ and UV/H_2_O_2_/Fe^2+^ processes, a series of experiments were conducted at initial concentration of 2.5, 5, 10, and 20 mg/L of [MG]_0_. For the UV/H_2_O_2_ process, the degradation efficiency was 100.00, 96.98, 84.91%, and 69.43% at 20 min of reaction time when [MG]_0_ = 2.5, 5, 10, and 20 mg/L of MG, respectively, while applying [H_2_O_2_]_0_ = 10 mM and pH = 6.0 (Figure 4). The degradation of MG followed *pseudo-first-order* kinetics, with the observed *pseudo-first-order* rate constant (*k*_obs_) of 0.1541, 0.1274, 0.0873, and 0.0524 min^−1^ at [MG]_0_ = 2.5, 5, 10, and 20 mg/L, respectively.

**FIGURE 5:**
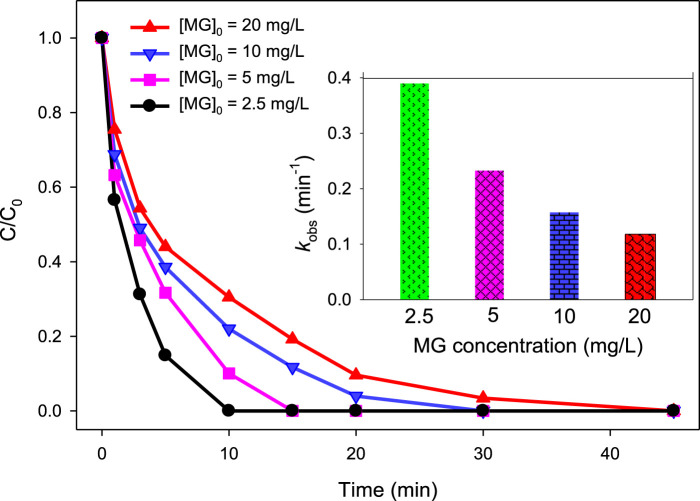
Effect of MG concentration on the UV/H_2_O_2_/Fe^2+^ process. [Fe^2+^]_0_ = 2.5 mg/L, [MG]_0_ = 2.5 mg/L, 5 mg/L, 10 mg/L, and 20 mg/L, [H_2_O_2_]_0_ = 10 mM, pH 3.0. The inset indicates the *k*_obs_ values at different MG concentrations.

For the UV/H_2_O_2_/Fe^2+^ process, the degradation efficiency was 100, 89.92, 77.99, and 69.49% at 10 min of treatment when the initial MG concentration was 2.5, 5, 10, and 20 mg/L, respectively (Figure 5), employing [Fe^2+^]_0_ = 2.5 mg/L, [H_2_O_2_]_0_ = 10 mM, and pH = 3.0. The *k*_obs_ values were found to be 0.3884, 0.2333, 0.1565, and 0.1180 min^‒1^ for [MG]_0_ = 2.5, 5, 10, and 20 mg/L, respectively.

These results clearly indicated that as the concentration of MG increases, the degradation efficiency decreases for both of the UV/H_2_O_2_ and UV/H_2_O_2_/Fe^2+^ processes. Since both these processes rely on the same reactive species, i.e., ^•^OH, and the source of ^•^OH (i.e., H_2_O_2_) is also the same in both processes, the reasons for the reduced degradation efficiency at higher MG concentration are also the same. The reduced degradation efficiency at a higher dye concentration is the solution’s elevated absorbance. This is particularly relevant because MG has a significant absorbance below 300 nm, which is the same range where H_2_O_2_ absorbs (Navarro et al., 2019). It means that the color produced by the MG dye acts as a filter for UV light and thereby reduces the penetration of light into the reaction mixture. As a result, at higher MG concentration, fewer photons are available to interact with the H_2_O_2_ molecule. Consequently, there will be lesser production of hydroxyl radicals, which are responsible for the degradation of dye molecules. By the same reason, the rate of regeneration of catalyst (Fe^2+^) *via* reaction (9) also decreases at higher MG concentration. Consequently, the rate of H_2_O_2_ activation by Fe^2+^ to produce ^•^OH *via* reaction (7) also decreases at higher MG concentration. Thus, the filter out of the UV light by the dye color resulted in reduced ^•^OH formation, which ultimately led to the lower degradation efficiency of MG in both UV/H_2_O_2_ and UV/H_2_O_2_/Fe^2+^ processes at higher MG concentration. Another possible reason of the lower degradation efficiency at higher MG concentration is the enhanced level of intermediate (degradation products of MG) molecules produced at higher MG concentration. As a result, the competition between MG and intermediate molecules for ^•^OH increases, and there are greater chances for ^•^OH to react with intermediate molecules rather than MG molecules when the level of intermediate molecules enhances at higher MG concentration, which led to lower degradation efficiency of MG.

Though the % degradation decreases with increase in MG concentration, the rate of degradation (the number of molecules undergo degradation per unit time) increases with increase in MG concentration. At higher MG concentration, a higher number of the dye molecules are exposed to reactive radicals, and hence, the chances of collisions between ^•^OH and MG molecules increase, which led to the higher degradation rate of MG (Khan et al., 2013; Rehman et al., 2018; Galindo et al., 2001). As a result, the rate of MG degradation was calculated to be 0.103, 0.217, 0.562, and 0.745 mg/L/min at 2.5, 5, 10, and 20 mg/L MG concentration, respectively, at 10 min of reaction time for the UV/H_2_O_2_ process. Similarly, for the UV/H_2_O_2_/Fe^2+^ process at 5 min of reaction time, the rate of degradation of MG was found to be 0.426, 0.770, 1.130, and 2.191 mg/L/min at 2.5, 5, 10, and 20 mg/L MG concentration, respectively.

Of note, % degradation is a relative term that refers to the number of molecules undergoing degradation related to the total number of molecules in the system. This relative quantity decreases with an increase in the target compound concentration. On the other hand, the rate of degradation is an absolute term which represents the actual number of molecules undergo degradation in the given specified time. It is not related to the total number of molecules. This absolute quantity increases with an increase in the target compound concentration.

#### 3.2.3 Effect of Fe^2+^ concentration on MG degradation by the UV/H_2_O_2_/Fe^2+^ process

**FIGURE 6:**
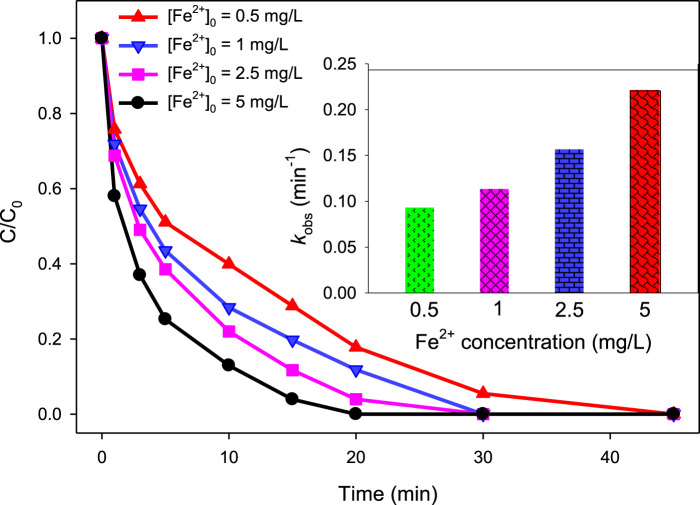
Effect of Fe^2+^ concentration on % degradation of MG in the UV/H_2_O_2_/Fe^2+^ process. [MG]_0_ = 10 mg/L, [H_2_O_2_]_0_ = 10 mM, pH 3.0. The inset indicates the relationship between *k*_obs_ and Fe^2+^ concentration.

To investigate the effect of different concentrations of Fe^2+^ on the degradation of MG by the UV/H_2_O_2_/Fe^2+^ process, a series of experiments were conducted using different initial concentrations of Fe^2+^ (i.e., 0.5, 1, 2.5, and 5 mg/L). Other conditions were kept constant at [H_2_O_2_]_0_ = 10 mM, [MG]_0_ = 10 mg/L, and pH = 3.0. The obtained results are depicted in Figure 6. The results indicated that the degradation efficiency increases with increasing the initial concentration of Fe^2+^ ([Fe^2+^]_0_). At [Fe^2+^]_0_ of 0.5, 1.0, 2.5, and 5.0 mg/L, the degradation efficiencies were 71.17, 80.25, 88.31, and 96.05% at 15 min, respectively. Similarly, the values of *k*_obs_ were found to be 0.093, 0.113, 0.1565, and 0.2204 min^−1^ at [Fe^2+^]_0_ = 0.5, 1.0, 2.5, and 5.0 mg/L, respectively. The increase in degradation of MG with an increase in [Fe^2+^]_0_ is due to the presence of Fenton-like reaction (Equation 7). Fe^2+^ triggers the activation of H_2_O_2_, leading to the formation of ^•^OH *via* an electron transfer process where electrons move from Fe^2+^ to H_2_O_2_ (De Laat and Gallard, 1999). Therefore, as the [Fe^2+^]_0_ increases, the rate of ^•^OH formation increases, which subsequently led to higher degradation efficiency of MG.

Further insight into the relationship between *k*_obs_ and [Fe^2+^]_0_ showed that the increase in *k*_obs_ with increase in [Fe^2+^]_0_ is not linear (Figure 6 inset). This non-linear relation is due to the ^•^OH scavenging effect of Fe^2+^ at a substantially higher concentration (Equation 11) (Hameed and Lee, 2009; Chen and Pignatello, 1997; Joseph et al., 2000).

O•H+Fe2+→Fe3++OH− k=4.3×108 M−1 s−1. (11)

#### 3.2.4 Effect of pH

**FIGURE 7:**
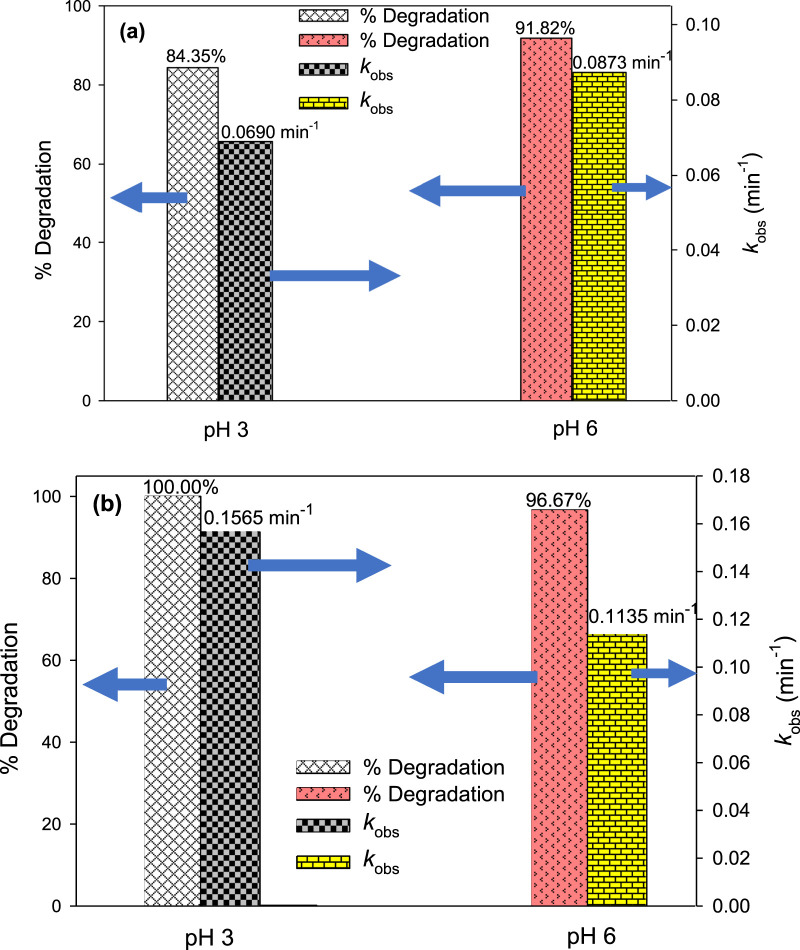
Effect of pH on % degradation and *k*_obs_ of MG by UV/H_2_O_2_ **(A)** and UV/H_2_O_2_/Fe^2+^ **(B)**. Experimental conditions: [MG]_0_ = 10 mg/L, [H_2_O_2_]_0_ = 10 mM, and [Fe^2+^]_0_ = 2.5 mg/L.

pH is a crucial factor that significantly impacts the effectiveness of AOPs by influencing the generation of ^•^OH and is consistently taken into account for the optimization of water treatment processes. To study the effect of pH on the degradation of MG by UV/H_2_O_2_ and UV/H_2_O_2_/Fe^2+^ processes and determine the optimal pH of the reaction mixture, experiments were carried out at pH = 3.0 and 6.0. For UV/H_2_O_2_, the concentrations of MG and H_2_O_2_ were kept constant at 10 mg/L and 10 mM, respectively. For UV/H_2_O_2_/Fe^2+^, the same concentrations of MG and H_2_O_2_ were used in addition to [Fe^2+^]_0_ = 2.5 mg/L. The results indicated that the degradation of MG was influenced by the pH of the solution (Figure 7). For the UV/H_2_O_2_ process, the degradation efficiency was higher at pH 6.0 compared to pH 3.0. Specifically, 91.82% MG degradation was observed at pH 6.0 compared to 84.35% at pH 3.0 after 30 min of irradiation. The *k*_obs_ value decreased from 0.0873 to 0.0690 min^‒1^ as the pH changed from 6.0 to 3.0 (Figure 7A). However, 100% degradation of MG was achieved after 60 min of treatment at both pH 3.0 and 6.0. Hence, the UV/H_2_O_2_ process shows higher degradation efficiency at pH 6.0 as compared to acidic pH. Iron-based processes such as Fenton and photo-Fenton are AOPs that demonstrate a strong pH dependence where acidity has been found as a favorable condition for the degradation of target compounds (Khan et al., 2013; Chan and Chu, 2003; Yong et al., 2015). Unlike the UV/H_2_O_2_ process, UV/H_2_O_2_/Fe^2+^ showed higher removal efficiency at pH 3.0 than at pH 6.0. The degradation of MG was found to be 100.00% and 96.67% after 30 min of treatment at pH 3.0 and pH 6.0, corresponding to *k*_obs_ of 0.1565 and 0.1135 min^‒1^, respectively (Figure 7B). A comparable pattern was noticed by Hassan et al. (2023) and Khan et al. (2013). Fe^2+^ has been found to have higher catalytic activity at acidic conditions for H_2_O_2_ activation to generate ^•^OH (Equation 7) (Khan et al., 2013; Chan and Chu, 2003). As the pH increases, iron undergoes precipitation in the form of Fe(OH)_3_ and hence, Fe^2+^ was not available to activate H_2_O_2_ for ^•^OH generation, thereby hindering the degradation efficiency of MG at pH 6.0 (Deb et al., 2023).

To better understand the efficiencies of the AOPs studied in this work in comparison with the similar AOPs reported previously on the degradation of MG, please refer to Table 1. Moreover, Table 2 summarizes the comparison of the efficiencies of different AOPs applied for the treatment of MG. Both these tables provide important information for enriching the existing knowledge on the removal of dyes by various AOPs.

**TABLE 1:** Comparison of MG removal by UV/H_2_O_2_ and UV/H_2_O_2_/Fe^2+^ processes studied in this work and those reported by other researchers.

Method	Concentration of malachite green (mg/L)	[H_2_O_2_]_0_	[Fe^2+^]_0_	pH	Time (min)	Degradation (%)	Reference
UV/H_2_O_2_	10	10 mM	----	6.0	60	100	This study
UV/H_2_O_2_/Fe^2+^	10	10 mM	2.5 mg/L	3.0	30	100	This study
UV/H_2_O_2_	10	300 mg/L	----	----	≈25–30	100	Modirshahla and Behnajady (2006)
UV/H_2_O_2_	25	1.5 mL of 33% H_2_O_2_/250 mL	----	5.6	50	96	Dar et al. (2023)
UV/H_2_O_2_/Fe^2+^	25	1.5 mL of 33% H_2_O_2_/250 mL	----	----	40	100	Dar et al. (2023)
UV/H_2_O_2_	100	12 mM	----	3.0	60	95	Ghime et al. (2019)
UV/H_2_O_2_/Fe^2+^	100	12 mM	60 ppm	3.0	60	98	Ghime et al. (2019)

**TABLE 2:** Comparison of different AOPs for the degradation of malachite green.

Method	Malachite green (mg/L)	Experimental condition	Time (min)	Degradation (%)	Reference
UV/H_2_O_2_	10	10 mM H_2_O_2_, pH 6.0	60	100	This study
UV/H_2_O_2_/Fe^2+^	10	10 mM H_2_O_2_, 2.5 mg/L Fe^2+^, pH 3.0		100	This study
TiO_2_ photocatalysis	100	0.6 g/L TiO_2_, UV light (32-W lamp)	60	95	Ghime et al. (2019)
TiO_2_ photocatalysis	10	0.5 g/L TiO_2_, UV-A light (9 W lamp)	40	100	Berberidou et al. (2007)
Sonolysis (under argon)	10	135 W ultrasound power, argon atmosphere	120	100	Berberidou et al. (2007)
Chemical oxidation with ozone (O_3_)	36.49	Ozone (0.5 g/h), pH 3.0, 5.0, and 7.0	110	96.74 at pH 3.0, 80.5 at pH 5.0, and 50.1 at pH 7.0	Mirila et al. (2018)
Electrochemical oxidation	20	I = 32 mA.cm^−2^, pH = 3, [Na_2_SO_4_] = 0.1 mol/L, boron-doped diamond (BDD)	60	98	Guenfoud et al. (2014)
Kissiris/Fe_3_O_4_/TiO_2_ with the glucose oxidase (GOx) enzyme	20	Initial glucose concentration = 20 mM, pH 5.5, temp. = 40°C, catalyst = 2 g	120	99	Elhami et al. (2015)

#### 3.2.5 Effect of chloride and nitrate ions

To investigate the effect of common inorganic ions on the degradation efficiency of MG by UV/H_2_O_2_ and UV/H_2_O_2_/Fe^2+^ processes, the degradation of MG was studied in the presence of chloride (Cl^−^) and nitrate ions (NO_3_^−^)- used as representative inorganic ions. It was found that both Cl^−^ and NO_3_^−^ slightly reduced the degradation efficiency of MG (Table 3). For the UV/H_2_O_2_ process, the degradation of MG decreased from 100.0% to 84.1% and 91.95% at 60 min of treatment in the presence of 5 mM each of Cl^−^ and NO_3_^−^, respectively, employing [MG]_0_ = 10 mg/L [H_2_O_2_]_0_ = 10 mM, and [pH]_0_ = 6. The *k*_obs_ was found to be 0.0873, 0.0415, and 0.0502 min^‒1^ in the presence of no scavenger, Cl^−,^ and NO_3_^−^, respectively (Table 3). Similarly, for the UV/H_2_O_2_/Fe^2+^ process, the degradation of MG decreased from 96.0% to 78.2% and 85.5% at 20 min of treatment in the presence of 5 mM each of Cl^−^ and NO_3_^−^, respectively, employing [MG]_0_ = 10 mg/L, [H_2_O_2_]_0_ = 10 mM, [Fe^2+^]_0_ = 2.5 mg/L, and [pH]_0_ = 3. The *k*_obs_ of MG by UV/H_2_O_2_/Fe^2+^ was found to be 0.1565, 0.0817, and 0.1016 min^‒1^ in the presence of no scavenger, Cl^−,^ and NO_3_^−^, respectively (Table 3).

**TABLE 3:** Effect of chloride and nitrate ions on % degradation and *k*_obs_ of MG by UV/H_2_O_2_ and UV/H_2_O_2_/Fe^2+^ processes. Experimental conditions: [MG]_0_ = 10 mg/L, [H_2_O_2_]_0_ = 10 mM, [Fe^2+^]_0_ = 2.5 mg/L. pH was 6.0 for UV/H_2_O_2_ and 3.0 for UV/H_2_O_2_/Fe^2+^.

Anion	UV/H_2_O_2_		UV/H_2_O_2_/Fe^2+^	
	% degradation at 60 min of treatment	*k*_obs_ (min^‒1^)	% degradation at 20 min of treatment	*k*_obs_ (min^‒1^)
No scavenger	100.00	0.0873	96.00	0.1565
Chloride ion (5 mM)	84.10	0.0415	78.18	0.0817
Nitrate ion (5 mM)	91.95	0.0502	85.46	0.1016

The reduction in the degradation efficiency of MG by Cl^−^ in both processes, i.e., UV/H_2_O_2_ and UV/H_2_O_2_/Fe^2+^, could possibly be attributed to the scavenging effect of Cl^−^ for ^•^OH. This is because the main reactive species in both of these processes is ^•^OH, as confirmed by the methanol scavenging test (data not shown). Chloride ions quench ^•^OH effectively in accordance of reactions Equation 12, 13 (Muruganandham and Swaminathan, 2004; Alshamsi et al., 2007).

Cl−+OH•⇌ClOH•−kf=4.3×109M−1 s−1,kb=6.1×109 M−1 s−1, (12)

$$\begin{array}{cc} \text{ClO}H^{•-}+{\;H}^{+}⇌Cl^{•}+H_{2}O & k_{f}=2.6\times 10^{10}\;M^{-1}\;s^{-1},k_{b}=3.6\times 10^{3}\;M^{-1}\;s^{-1}. \end{array}$$ (13)

The slight decrease in the removal efficiency of MG by Cl^−^ may possibly be due to the reversibility of reaction Equation 12, which re-produces the ^•^OH *via* backward reaction with a little higher rate constant (*k*_*b*_) than its scavenging *via* forward reaction (*k*_*f*_). Moreover, the presence of Fe^2+^ in UV/H_2_O_2_/Fe^2+^, could also potentially contribute to the lower removal efficiency of MG by Cl^−^ (reaction (Equation 14)) (Ali et al., 2018; Malik and Saha, 2003).

$${\text{ClOH}}^{•-}+{\text{Fe}}^{2+}\to {\text{Cl}}^{-}+{\text{OH}}^{-}+{\text{Fe}}^{3+}.$$ (14)

Nitrate ions (NO_3_^−^) were found to have a less retarding effect than Cl^−^ on the degradation of MG by both UV/H_2_O_2_ and UV/H_2_O_2_/Fe^2+^ processes. This could possibly be due to the low reactivity of NO_3_^−^ with ^•^OH (Rehman et al., 2018).

### 3.3 Identification of degradation products and possible degradation pathways

The DPs of MG produced during UV/H_2_O_2_ treatment were identified by GC-MS. Prior to discussing the experimentally detected DPs of MG, it is highly useful to find the potential reactive sites of MG where ^•^OH could initially attack *via* addition or hydrogen abstraction reactions. For this purpose, the density functional theory (DFT) calculations were performed using the HF/3–21 g basis set for optimization and B3LYP/6–311 g basis set for energy calculations. The DFT calculations were performed using the Gaussian 09 program to find out the FEDs and point charges of the MG atoms to predict the positions in the MG molecule where ^•^OH can easily undergo addition or hydrogen abstraction reactions. The calculated values of FEDs and point charges of the MG are given in Table 4. It has been reported that atoms having larger values of (FED^2^_HOMO_ + FED^2^_LUMO_) are more likely to be attacked by the ^•^OH *via* addition reaction/pathway (An et al., 2015). On the other hand, ^•^OH could preferably abstract hydrogen from atoms having more positive charge. Based on this information, the ^•^OH could initially react with C7 atom *via* the addition reaction due to its large (FED^2^_HOMO_ + FED^2^_LUMO_) value (An et al., 2015; Rehman et al., 2018). The next preferable sites of ^•^OH addition reactions could be the N20 and N23 atoms followed by the C4 and C8 atoms. The highest point charge values are possessed by C1 and C11 atoms due to their attachment with the electronegative N atoms. However, both of these C atoms bear no hydrogen atom to be abstracted by ^•^OH. Similarly, the carbon atoms bearing the next higher value of point charge, i.e., C7, also lack hydrogen atoms to be abstracted by ^•^OH. The same is the case with C14, which has the next higher value of point charge. Therefore, the possible hydrogen abstraction sites are proposed to be C3 and C9 as well as C5 and C13, which have the lowest negative values (i.e., relatively large values) of point charge among the atoms bearing hydrogen atoms.

**TABLE 4:** Frontier electron densities and point charges of MG atoms calculated by the Gaussian 09 program.

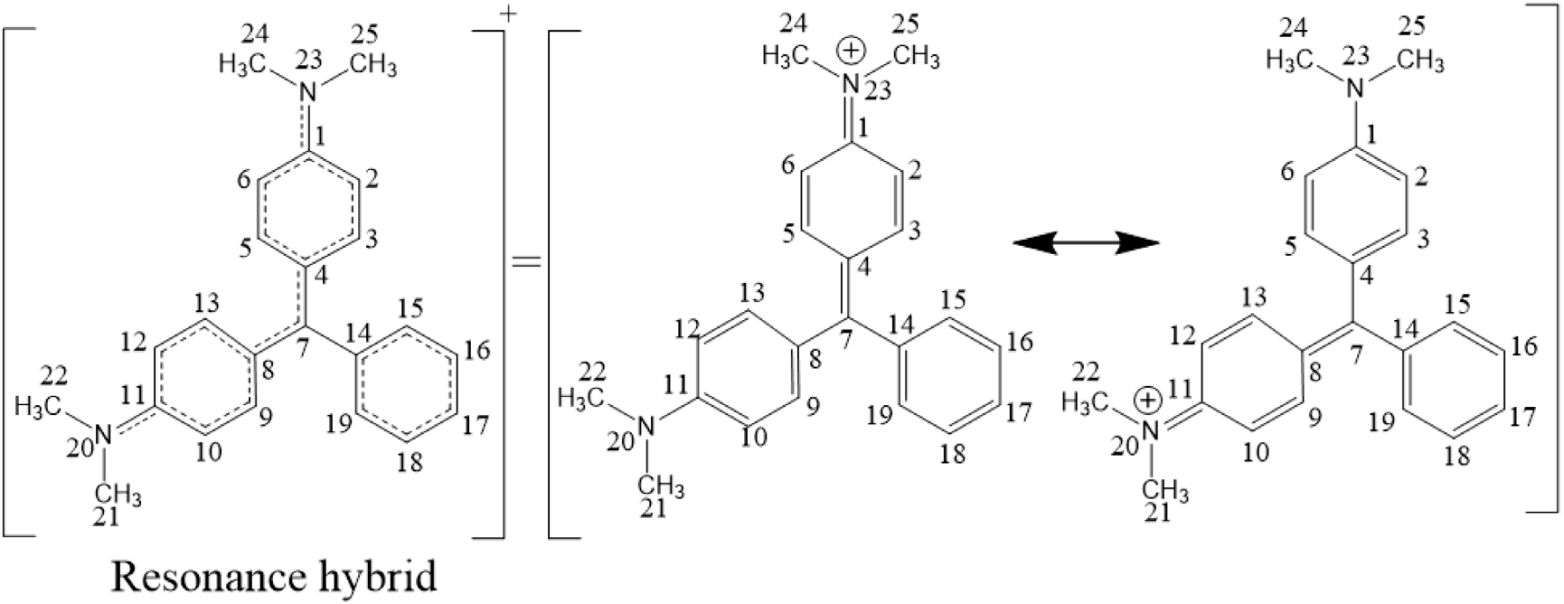					
Atom	FED^2^_HOMO_ + FED^2^_LUMO_	Point charge	Atom	FED^2^_HOMO_ + FED^2^_LUMO_	Point charge
C1	0.0590	0.23732	C14	0.0236	−0.09591
C2	0.0452	−0.25008	C15	0.0408	−0.16964
C3	0.0578	−0.12135	C16	0.0066	−0.18682
C4	0.0774	−0.13303	C17	0.0288	−0.15825
C5	0.0567	−0.12959	C18	0.0066	−0.18681
C6	0.0480	−0.25203	C19	0.0408	−0.16964
C7	0.2168	0.15527	N20	0.1724	−0.36351
C8	0.0774	−0.13303	C21	0.0019	−0.36622
C9	0.0578	−0.12135	C22	0.0019	−0.36616
C10	0.0452	−0.25008	N23	0.1723	−0.36351
C11	0.0590	0.23732	C24	0.0019	−0.36616
C12	0.0479	−0.25203	C25	0.0019	−0.36622
C13	0.0567	−0.12960			

**Scheme 1:**
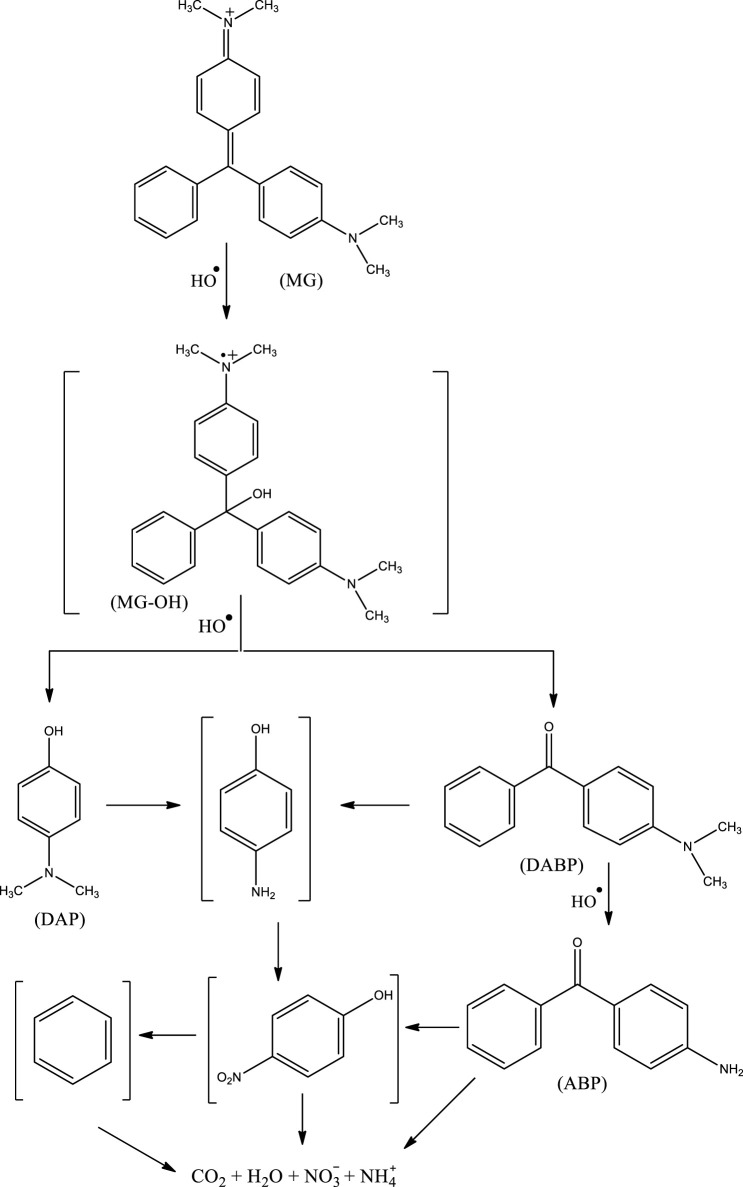
Proposed degradation mechanism of malachite green by the UV/H_2_O_2_ process.

In the present work, three DPs of MG were identified, namely: (a) 4-dimethylamino-benzophenone (DABP), (b) 4-amino-benzophenone (ABP), and (c) 4-dimethylamino-phenol (DAP) (Scheme 1). The hydroxyl radical could possibly attack the central carbon atom of MG, leading to the formation of a hydroxylated reactive cationic radical (MG-OH)—not detected in the present study but is supposed to be a tentative degradation product based on the DFT calculations (Berberidou et al., 2007). The subsequent demethylation and further oxidation by ^•^OH finally led to the formation of DAP and DABP (Berberidou et al., 2007). The demethylation of DABP—a common reaction of MG—could then lead to the formation of ABP (Berberidou et al., 2007). The mentioned three DPs have also been detected by other researchers while studying the degradation of MG by AOPs (Berberidou et al., 2007; Milano et al., 1995; Xie et al., 2001; Chen et al., 2002; Ju et al., 2009). Moreover, it has been previously reported that the cleavage of benzophenone generally leads to the formation of benzene and benzaldehyde (Berberidou et al., 2007). The formation of amino-benzene from MG and then its conversion to nitro-benzene by ^•^OH have also been reported (Berberidou et al., 2007). The attack of ^•^OH on lower-molecular weight aromatic DPs such as amino-benzene, nitro-benzene, benzaldehyde, and benzene further leads to the formation of lower-molecular weight organic acids, which subsequently lead to the formation of CO_2_, H_2_O, nitrate, nitrite, and/or ammonium ions (Xie et al., 2001; Ju et al., 2009). The present study suggests that treatment of MG-containing water by HR-AOPs not only leads to its complete degradation but also leads to its complete mineralization—albeit slowly.

### 3.4 Toxicity evaluation

The ultimate goal of a water treatment technology is to achieve clean and pure water, i.e., toxicant-free water. To check whether the toxicity of the treating mixture reduces during the degradation of MG or not, the aquatic toxicity of the detected DPs toward fish, daphnia, and green algae was determined using the ECOSAR program (Rehman et al., 2023; Iqbal et al., 2024a; Iqbal et al., 2024b; Khan et al., 2023) (Table 5). Surprisingly, all the three detected DPs were found to have higher toxicity (both acute and chronic) than the parent MG. Therefore, it can be concluded that the researchers should not just focus on the removal of target compound while dealing with the removal of dyes. Rather, the toxicity of the treated solution should be determined before, during, and after the treatment in order to ensure the reduction in toxicity of the treated solution. Moreover, the treatment time should be prolonged such that the treatment technology not only degrades/removes the target pollutant but also the DPs, i.e., until complete mineralization is achieved.

**TABLE 5:** Aquatic toxicity of malachite green and its identified DPs (unit = mg L^−1^).

Compound	Acute toxicity			Chronic toxicity		
	Fish (LC_50_)	Daphnia (LC_50_)	Green algae (EC_50_)	Fish (ChV)	Daphnia (ChV)	Green algae (ChV)
MG	3,230	1,640	774	277	118	158
DABP	12.0	7.67	9.48	1.35	1.05	3.26
ABP	15.1	1.79	4.48	0.138	0.022	1.11
DAP	442	236	137	40.2	19.4	31.2

## 4 Conclusion

The present study investigated the degradation of MG by UV/H_2_O_2_ and UV/H_2_O_2_/Fe^2+^ processes. MG was found to be resistant toward direct photolysis as only 12.35% MG degradation was achieved by direct photolysis at pH 6.0 after 60 min of treatment. However, the combination of H_2_O_2_ with UV light accelerated the degradation of MG, achieving 100% degradation of MG at 10 mM H_2_O_2_ concentration when treated for 60 min, suggesting the higher reactivity of MG with ^•^OH. Further enhancement in the degradation efficiency of MG by UV/H_2_O_2_ was observed by adding Fe^2+^ ion—an effective catalyst for H_2_O_2_ activation—as 100% degradation of MG was observed at [Fe^2+^]_0_ = 2.5 mg/L and 30 min of treatment. The higher concentration of MG was found to have a detrimental effect on its degradation by both UV/H_2_O_2_ and UV/H_2_O_2_/Fe^2+^ processes. However, increasing concentrations of H_2_O_2_ and Fe^2+^ were found to have a positive effect on MG degradation. For the UV/H_2_O_2_ process, a higher removal efficiency of MG was observed at pH 6.0 as compared to at pH 3.0. Nitrate (NO_3_^−^) and chloride ions (Cl^−^) have negatively impacted the MG degradation by both UV/H_2_O_2_ and UV/H_2_O_2_/Fe^2+^ processes. However, NO_3_^−^ showed less retarding effect than Cl^−^, possibly due to the lower reactivity of NO_3_^−^ with ^•^OH than that of Cl^−^. Based on the GC-MS analysis, three degradation products (DPs) of MG were identified, namely: (a) 4-dimethylamino-benzophenone (DABP), (b) 4-amino-benzophenone (ABP), and (c) 4-dimethylamino-phenol (DAP). The computational aquatic toxicity study toward three aquatic organisms (fish, daphnia, and green algae) showed that all of the detected DPs are more toxic than MG. The present study revealed that MG can effectively be degraded by hydroxyl radical-based AOPs; however, researchers should also ensure the complete removal of its DPs so that the toxicity of the treated water may not increase due to the formation of toxic DPs.

## Data Availability

The original contributions presented in the study are included in the article; further inquiries can be directed to the corresponding authors.

## References

[B1] Ali F. Khan J. A. Shah N. S. Sayed M. Khan H. M. (2018). Carbamazepine degradation by UV and UV-assisted AOPs: kinetics, mechanism and toxicity investigations. Process Saf. Environ. Prot. 117, 307–314. 10.1016/j.psep.2018.05.004

[B2] Alshamsi F. A. Albadwawi A. S. Alnuaimi M. M. Rauf M. A. Ashraf S. S. (2007). Comparative efficiencies of the degradation of Crystal Violet using UV/hydrogen peroxide and Fenton's reagent. Dyes Pigm 74, 283–287. 10.1016/j.dyepig.2006.02.016

[B3] Alsukaibi A. K. (2022). Various approaches for the detoxification of toxic dyes in wastewater. Processes 10 (10), 1968. 10.3390/pr10101968

[B4] An T. An J. Gao Y. Li G. Fang H. Song W. (2015). Photocatalytic degradation and mineralization mechanism and toxicity assessment of antivirus drug acyclovir: experimental and theoretical studies. Appl. Catal. B Environ. 164, 279–287. 10.1016/j.apcatb.2014.09.009

[B5] Arora S. (2014). Textile dyes: it’s impact on environment and its treatment. J. Bioremed. Biodeg 5 (3), 1. 10.4172/2155-6199.1000e146

[B6] Berberidou C. Poulios I. Xekoukoulotakis N. P. Mantzavinos D. (2007). Sonolytic, photocatalytic and sonophotocatalytic degradation of malachite green in aqueous solutions. Appl. Catal. B Environ. 74, 63–72. 10.1016/j.apcatb.2007.01.013

[B7] Chan K. Chu W. (2003). Modeling the reaction kinetics of Fenton’s process on the removal of atrazine. Chemosphere 51, 305–311. 10.1016/s0045-6535(02)00812-3 12604082

[B8] Chen F. He J. Zhao J. Yu J. C. (2002). Photo-Fenton degradation of malachite green catalyzed by aromatic compounds under visible light irradiation. New J. Chem. 26, 336–341. 10.1039/b107404k

[B9] Chen R. Pignatello J. (1997). Role of quinone intermediates as electron shuttles in Fenton and photoassisted Fenton oxidations of aromatic compounds. Environ. Sci. Technol. 31, 2399–2406. 10.1021/es9610646

[B10] Chen Y. Hu C. Qu J. Yang M. Chemistry P. A. (2008). Photodegradation of tetracycline and formation of reactive oxygen species in aqueous tetracycline solution under simulated sunlight irradiation. J. Photochem. Photobiol. A Chem. 197, 81–87. 10.1016/j.jphotochem.2007.12.007

[B11] Dar A. Anwar J. Munir A. (2023). Photocatalytic degradation of malachite green dye with UV/H2O2 system in presence of transition metal ions. J. Chem. Soc. Pak. 45 (6), 302. 10.52568/001284/jcsp/45.04.2023

[B12] Deb A. Rumky J. Sillanpää M. (2023). “Fenton, photo-Fenton, and electro-Fenton systems for micropollutant treatment processes,” in Advanced oxidation processes for micropollutant remediation. Editors Khalid M. Park Y. Karri R. R. Walvekar R. (Boca Raton, FL: CRC press, Taylor & Francis group), 157–185. 10.1201/9781003247913-8

[B13] De Laat J. Gallard H. (1999). Catalytic decomposition of hydrogen peroxide by Fe (III) in homogeneous aqueous solution: mechanism and kinetic modeling. Environ. Sci. Technol. 33, 2726–2732. 10.1021/es981171v

[B14] Elhami V. Karimi A. Aghbolaghy M. (2015). Preparation of heterogeneous bio-Fenton catalyst for decolorization of Malachite Green. J. Taiwan Inst. Chem. E. 56, 154–159. 10.1016/j.jtice.2015.05.006

[B15] Fast S. A. Gude V. G. Truax D. D. Martin J. Magbanua B. S. (2017). A critical evaluation of advanced oxidation processes for emerging contaminants removal. Environ. Process. 4, 283–302. 10.1007/s40710-017-0207-1

[B16] Galindo C. Jacques P. Kalt A. P A. (2001). Photochemical and photocatalytic degradation of an indigoid dye: a case study of acid blue 74 (AB74). J. Photochem. Photobiol. A Chem. 141, 47–56. 10.1016/s1010-6030(01)00435-x

[B17] Garrido-Cardenas J. A. Esteban-García B. Agüera A. Sánchez-Pérez J. A. Manzano-Agugliaro F. (2020). Wastewater treatment by advanced oxidation process and their worldwide research trends. Int. J. Environ. Res. Public Health 17 (1), 170. 10.3390/ijerph17010170 PMC6981484 31881722

[B18] Gharavi-Nakhjavani M. S. Niazi A. Hosseini H. Aminzare M. Dizaji R. Tajdar-Oranj B. (2023). Malachite green and leucomalachite green in fish: a global systematic review and meta-analysis. Environ. Sci. Pollut. Res. 30 (17), 48911–48927. 10.1007/s11356-023-26372-z 36920616

[B19] Ghime D. Goru P. Ojha S. Ghosh P. (2019). Oxidative decolorization of a malachite green oxalate dye through the photochemical advanced oxidation processes. Glob. Nest J. 21 (2), 195. 10.30955/gnj.003000

[B20] Gonzalez M. G. Oliveros E. Wörner M. Braun A. M. (2004). Vacuum-ultraviolet photolysis of aqueous reaction systems. J. Photochem. Photobiol. C. Photochem. Rev. 5, 225–246. 10.1016/j.jphotochemrev.2004.10.002

[B21] Gopinathan R. Kanhere J. Banerjee J. (2015). Effect of malachite green toxicity on non-target soil organisms. Chemosphere 120, 637–644. 10.1016/j.chemosphere.2014.09.043 25462308

[B22] Guenfoud F. Mokhtari M. Akrout H. (2014). Electrochemical degradation of malachite green with BDD electrodes: effect of electrochemical parameters. Diam. Relat. Mater. 46, 8–14. 10.1016/j.diamond.2014.04.003

[B23] Hameed B. Lee T. (2009). Degradation of malachite green in aqueous solution by Fenton process. J. Hazard. Mater. 164, 468–472. 10.1016/j.jhazmat.2008.08.018 18804913

[B24] Hassan A. F. Mustafa A. Esmail G. Awad A. (2023). Adsorption and photo-fenton degradation of methylene blue using nanomagnetite/potassium carrageenan bio-composite beads. Arab. J. Sci. Eng. 48, 353–373. 10.1007/s13369-022-07075-y

[B25] Hu J. Chen S. Liang X. (2022). Heterogeneous catalytic oxidation for the degradation of aniline in aqueous solution by persulfate activated with CuFe_2_O_4_/activated carbon catalyst. ChemistrySelect 7, e202201241. 10.1002/slct.202201241

[B26] Imoberdorf G. Mohseni M. (2011). Degradation of natural organic matter in surface water using vacuum-UV irradiation. J. Hazard. Mater. 186, 240–246. 10.1016/j.jhazmat.2010.10.118 21122985

[B27] Iqbal J. Shah N. S. Ali Khan J. Ibrahim A. Masood Pirzada B. Naushad M. (2024a). Visible light driven ZnFe_2_O_4_ for the degradation of oxytetracycline in the presence of HSO_5_ ^−^ at semi-pilot scale and additional H_2_ production. Chem. Eng. J. 498, 155402. 10.1016/j.cej.2024.155402

[B28] Iqbal J. Shah N. S. Ali Khan J. Naushad M. Boczkaj G. Jamil F. (2024b). Pharmaceuticals wastewater treatment via different advanced oxidation processes: reaction mechanism, operational factors, toxicities, and cost evaluation – a review. Sep. Purif. Technol. 347, 127458. 10.1016/j.seppur.2024.127458

[B29] Islam M. Kumar S. Saxena N. Nafees A. (2023). Photocatalytic degradation of dyes present in industrial effluents: a review. ChemistrySelect 8, e202301048. 10.1002/slct.202301048

[B30] Jiad M. M. Abbar A. H. (2023). Efficient wastewater treatment in petroleum refineries: hybrid electro-Fenton and photocatalysis (UV/ZnO) process. Chem. Eng. Res. Des. 200, 431–444. 10.1016/j.cherd.2023.10.050

[B31] Joseph J. M. Destaillats H. Hung H.-M. Hoffmann M. R. (2000). The sonochemical degradation of azobenzene and related azo dyes: rate enhancements via Fenton's reactions. J. Phys. Chem. A 104, 301–307. 10.1021/jp992354m

[B32] Ju Y. Yang S. Ding Y. Sun C. Gu C. He Z. (2009). Microwave-enhanced H_2_O_2_-based process for treating aqueous malachite green solutions: intermediates and degradation mechanism. J. Hazard. Mater. 171, 123–132. 10.1016/j.jhazmat.2009.05.120 19573984

[B33] Kant R. (2012). Textile dyeing industry an environmental hazard. Nat. Sci. 4 (1), 22–26. 10.4236/ns.2012.41004

[B34] Katheresan V. Kansedo J. Lau S. Y. (2018). Efficiency of various recent wastewater dye removal methods: a review. J. Environ. Chem. Eng. 6 (4), 4676–4697. 10.1016/j.jece.2018.06.060

[B35] Khan I. Saeed K. Ali N. Khan I. Zhang B. Sadiq M. (2020a). Heterogeneous photodegradation of industrial dyes: an insight to different mechanisms and rate affecting parameters. J. Environ. Chem. Eng. 8 (5), 104364. 10.1016/j.jece.2020.104364

[B36] Khan J. A. He X. Khan H. M. Shah N. S. Dionysiou D. D. (2013). Oxidative degradation of atrazine in aqueous solution by UV/H_2_O_2_/Fe^2+^, UV/S_2_O_8_ ^2−^/Fe^2+^ and UV/HSO_5_ ^−^/Fe^2+^ processes: a comparative study. Chem. Eng. J. 218, 376–383. 10.1016/j.cej.2012.12.055

[B37] Khan J. A. He X. Shah N. S. Khan H. M. Hapeshi E. Fatta-Kassinos D. (2014). Kinetic and mechanism investigation on the photochemical degradation of atrazine with activated H_2_O_2_, S_2_O_8_ ^2−^ and HSO_5_ ^−^ . Chem. Eng. J. 252, 393–403. 10.1016/j.cej.2014.04.104

[B38] Khan J. A. He X. Shah N. S. Sayed M. Khan H. M. Dionysiou D. D. (2017). Degradation kinetics and mechanism of desethyl-atrazine and desisopropyl-atrazine in water with ^•^OH and SO_4_ ^•−^ based-AOPs. Chem. Eng. J. 325, 485–494. 10.1016/j.cej.2017.05.011

[B39] Khan J. A. Sayed M. Shah N. S. Khan S. Khan A. A. Sultan M. (2023). Synthesis of N-doped TiO_2_ nanoparticles with enhanced photocatalytic activity for 2,4-dichlorophenol degradation and H_2_ production. J. Environ. Chem. Eng. 11, 111308. 10.1016/j.jece.2023.111308

[B40] Khan J. A. Sayed M. Shah N. S. Khan S. Zhang Y. Boczkaj G. (2020b). Synthesis of eosin modified TiO_2_ film with co-exposed {001} and {101} facets for photocatalytic degradation of para-aminobenzoic acid and solar H_2_ production. Appl. Catal. B Environ. 265, 118557. 10.1016/j.apcatb.2019.118557

[B41] Kishor R. Purchase D. Saratale G. D. Saratale R. G. Ferreira L. F. R. Bilal M. (2021). Ecotoxicological and health concerns of persistent coloring pollutants of textile industry wastewater and treatment approaches for environmental safety. J. Environ. Chem. Eng. 9 (2), 105012. 10.1016/j.jece.2020.105012

[B42] Lal R. Gour T. Dave N. Singh N. Yadav J. Khan A. (2024). Green route to fabrication of Semal-ZnO nanoparticles for efficient solar-driven catalysis of noxious dyes in diverse aquatic environments. Front. Chem. 12, 1370667. 10.3389/fchem.2024.1370667 38817442 PMC11137298

[B43] Lanjwani M. F. Tuzen M. Khuhawar M. Y. Saleh T. A. (2024). Trends in photocatalytic degradation of organic dye pollutants using nanoparticles: a review. Inorg. Chem. Commun. 159, 111613. 10.1016/j.inoche.2023.111613

[B44] Malik P. K. Saha S. K. (2003). Oxidation of direct dyes with hydrogen peroxide using ferrous ion as catalyst. Sep. Purif. Technol. 31, 241–250. 10.1016/s1383-5866(02)00200-9

[B45] Milano J. C. Loste-Berdot P. Vernet J. L. (1995). Photooxydation du Vert de Malachite en Milieu Aqueux en Presence de Peroxyde D'Hydrogene: Cinetique et Mecanisme Photooxidation of Malachite Green in Aqueous Medium in the Presence of Hydrogen Peroxide: Kinetic and Mechanism. Environ. Technol. 16, 329–341. 10.1080/09593331608616275

[B46] Mirila D. C. Pîrvan M. Ș. Platon N. Georgescu A. M. Zichil V. Nistor I. D. (2018). Total mineralization of malachite green dye by advanced oxidation processes. Acta Chem. Iasi 26, 263–280. 10.2478/achi-2018-0017

[B47] Mishra S. Chowdhary P. Bharagava R. N. (2019). “Conventional methods for the removal of industrial pollutants, their merits and demerits,” in Emerging and eco-friendly approaches for waste management. Editors Bharagava R. N. Chowdhary P. (Springer), 1–31.

[B48] Modirshahla N. Behnajady M. (2006). Photooxidative degradation of Malachite Green (MG) by UV/H_2_O_2_: influence of operational parameters and kinetic modeling. Dyes Pigm 70, 54–59. 10.1016/j.dyepig.2005.04.012

[B49] Muruganandham M. Swaminathan M. (2004). Photochemical oxidation of reactive azo dye with UV-H_2_O_2_ process. Dyes Pigm 62, 269–275. 10.1016/j.dyepig.2003.12.006

[B50] Nasuha N. Hameed B. Okoye P. (2021). Dark-Fenton oxidative degradation of methylene blue and acid blue 29 dyes using sulfuric acid-activated slag of the steel-making process. J. Environ. Chem. Eng. 9, 104831. 10.1016/j.jece.2020.104831

[B51] Navarro P. Zapata J. P. Gotor G. Gonzalez-Olmos R. Gómez-López V. (2019). Degradation of malachite green by a pulsed light/H_2_O_2_ process. Water Sci. Technol. 79, 260–269. 10.2166/wst.2019.041 30865597

[B52] Oh W. D. Dong Z. Lim T. T. (2016). Generation of sulfate radical through heterogeneous catalysis for organic contaminants removal: current development, challenges and prospects. Appl. Catal. B Environ. 194, 169–201. 10.1016/j.apcatb.2016.04.003

[B53] Oladoye P. O. Ajiboye T. O. Wanyonyi W. C. Omotola E. O. Oladipo M. E. (2023). Insights into remediation technology for malachite green wastewater treatment. Water Sci. Technol. 16 (3), 261–270. 10.1016/j.wse.2023.03.002

[B54] Rauf M. A. Ali L. Sadig M. S. Ashraf S. S. Hisaindee S. (2016). Comparative degradation studies of Malachite Green and Thiazole Yellow G and their binary mixture using UV/H_2_O_2_ . Desalin. Water Treat. 57, 8336–8342. 10.1080/19443994.2015.1017745

[B55] Rehman F. Parveen N. Iqbal J. Sayed M. Shah N. S. Ansar S. (2023). Potential degradation of norfloxacin using UV-C/Fe^2+^/peroxides-based oxidative pathways. J. Photochem. Photobiol. A Chem. 435, 114305. 10.1016/j.jphotochem.2022.114305

[B56] Rehman F. Sayed M. Khan J. A. Shah N. S. Khan H. M. Dionysiou D. D. (2018). Oxidative removal of brilliant green by UV/S_2_O_8_ ^2‒^, UV/HSO_5_ ^‒^ and UV/H_2_O_2_ processes in aqueous media: a comparative study. J. Hazard. Mater. 357, 506–514. 10.1016/j.jhazmat.2018.06.012 30008383

[B57] Sepúlveda M. Musiał J. Saldan I. Chennam P. K. Rodriguez-Pereira J. Sopha H. (2024). Photocatalytic degradation of naproxen using TiO_2_ single nanotubes. Front. Environ. Chem. 5, 1373320. 10.3389/fenvc.2024.1373320

[B58] Shah N. S. He X. Khan J. A. Khan H. M. Boccelli D. L. Dionysiou D. D. (2015). Comparative studies of various iron-mediated oxidative systems for the photochemical degradation of endosulfan in aqueous solution. J. Photochem. Photobiol. A Chem. 306, 80–86. 10.1016/j.jphotochem.2015.03.014

[B59] Sharma J. Sharma S. Soni V. (2023). Toxicity of malachite green on plants and its phytoremediation: a review. Regional Stud. Mar. Sci. 62, 102911. 10.1016/j.rsma.2023.102911

[B60] Singh K. Arora S. (2011). Removal of synthetic textile dyes from wastewaters: a critical review on present treatment technologies. Crit. Rev. Environ. Sci. Technol. 41, 807–878. 10.1080/10643380903218376

[B61] Sivaraman C. Vijayalakshmi S. Leonard E. Sagadevan S. Jambulingam R. (2022). Current developments in the effective removal of environmental pollutants through photocatalytic degradation using nanomaterials. Catalysts 12 (5), 544. 10.3390/catal12050544

[B62] Slama H. B. Chenari Bouket A. Pourhassan Z. Alenezi F. N. Silini A. Cherif-Silini H. (2021). Diversity of synthetic dyes from textile industries, discharge impacts and treatment methods. Appl. Sci. 11 (14), 6255. 10.3390/app11146255

[B63] Thao T. T. P. Nguyen-Thi M. L. Chung N. D. Ooi C. W. Park S. M. Lan T. T. (2023). Microbial biodegradation of recalcitrant synthetic dyes from textile-enriched wastewater by Fusarium oxysporum. Chemosphere 325, 138392. 10.1016/j.chemosphere.2023.138392 36921772

[B64] Tkaczyk A. Mitrowska K. Posyniak A. (2020). Synthetic organic dyes as contaminants of the aquatic environment and their implications for ecosystems: a review. Sci. Total Environ. 717, 137222. 10.1016/j.scitotenv.2020.137222 32084689

[B65] Wei Y. Wang C. Liu D. Jiang L. Chen X. Li H. (2020). Photo-catalytic oxidation for pyridine in circumneutral aqueous solution by magnetic Fe-Cu materials activated H_2_O_2_ . Chem. Eng. Res. Des. 163, 1–11. 10.1016/j.cherd.2020.08.007

[B66] Xie Y. Wu K. Chen F. He J. Zhao J. (2001). Investigation of the intermediates formed during the degradation of malachite green in the presence of Fe^3+^ and H_2_O_2_ under visible irradiation. Res. Chem. Intermed. 27, 237–248. 10.1163/156856701300356455

[B67] Yong L. Zhanqi G. Yuefei J. Xiaobin H. Cheng S. Shaogui Y. (2015). Photodegradation of malachite green under simulated and natural irradiation: kinetics, products, and pathways. J. Hazard. Mater. 285, 127–136. 10.1016/j.jhazmat.2014.11.041 25497025

